# IGRA as a Predictive Factor of Silent Pulmonary Changes in Individuals Following Exposure to Tuberculosis

**DOI:** 10.1007/s00408-014-9637-y

**Published:** 2014-08-17

**Authors:** Tomasz Targowski, Sylwia Chelstowska, Tadeusz Plusa

**Affiliations:** 1 Department of Internal Medicine, Pneumonology and Allergology, Military Institute of Medicine, Ul. Szaserów 128, 04-141 Warsaw, Poland; 2 Department of Internal Medicine and Hematology, Military Institute of Medicine, Warsaw, Poland

**Keywords:** Tuberculosis, IGRA, LTBI

## Abstract

**Objectives:**

We conducted a study on usefulness of the tuberculin skin test (TST) and the Quantiferon-TB Gold IT (QFT) tests as predictors of radiological changes after contact with tuberculosis.

**Materials and Methods:**

The study group consisted of TB-exposed HCWs working in the Military Institute of Medicine (Warsaw, Poland). The usefulness of TST, QFT, and a combination of both tests was assessed for prediction of silent radiological findings.

**Results:**

83 previously TB-exposed participants were recruited. None of the participants had a history of active tuberculosis. Positive TST results were reported in 72 (86.8 %) participants, and positive QFTs were observed in 27 (32.5 %) cases. Chest radiographs revealed 23 findings specific for non-active tuberculosis in 18 (21.7 %) participants. The results of the QFTs were associated with the highest negative predictive value, positive predictive value, and positive likelihood ratio of silent chest X-ray findings suggestive of latent tuberculosis infection. Positive QFT was the only statistically significant variable that increases the odds ratio (OR–8.3) of the presence of typical of tuberculosis radiological changes in the lung.

**Conclusion:**

A positive QFT result in an individual with no TB history who was exposed to tuberculosis in the past is associated with a significantly higher risk of clinically silent parenchymal lesions in lungs suggestive of previous tuberculosis.

## Introduction

Currently, two methods are available for the evaluation of contact with tuberculosis (TB), a tuberculin skin test (TST) and interferon-gamma release assays (IGRAs). Both of them are indirect methods of latent tuberculosis infection (LTBI) diagnosis because they only assess the immune system response to the *Mycobacterium tuberculosis* antigens. Thus, it is difficult to judge whether the positive results of these tests are evidence of actual TB bacteria presence in the body or merely an expression of the “immunological memory” of the immune system [1, 2]. Healthcare workers appeared as subpopulations at a higher risk of tuberculosis infection than the general population with no occupational exposure [3–7]. We decided to conduct a study on usefulness of the TST and one of the IGRAs—Quantiferon-TB Gold IT (QFT)—for the evaluation of predictive value of radiological changes following a contact with the index case in asymptomatic individuals with no history of tuberculosis.

## Materials and Methods

The study was approved by the Ethics Committee of the Military Institute of Medicine (Warsaw, Poland). The screening group consisted of 363 volunteers working in the Military Institute of Medicine in Warsaw (Poland). The main inclusion criterion was a known contact with tuberculosis in the past. Each participant signed the informed consent form and filled out a questionnaire concerning the socio-economic, occupational, and medical information as well as a history of the BCG vaccination and contact with tuberculosis in the past. Tuberculosis contacts around the index cases were classified according to the degree of exposure defined by the European consensus [1]. The exclusion criteria for the participants were as follows: any history of active tuberculosis or radiological pulmonary findings suggestive of tuberculosis or any new contact with tuberculosis from the date of the first contact to the date of inclusion into the study, any history of acute infection symptoms, any history or symptoms of chronic infection, any vaccination during 6 weeks prior to the study, HIV infection, pregnancy, treatment with steroids or any other immunosuppressive agents prior to the study, any history of gastrointestinal or lung resection, diabetes, sarcoidosis, rheumatoid arthritis, silicosis, any malignancy, chronic hepatic or renal failure, and indeterminate result of QFT. Moreover, an eligibility condition was a normal chest X-ray scan obtained within 2 months to 2 years since the contact with the index case.

All patients who met the inclusion/exclusion criteria were subjected to posteroanterior and lateral chest radiography; when the presence of radiological changes was doubtful, a chest CT was performed. The radiological findings were assessed independently by an experienced radiologist and a lung disease specialist. For the assessment of any doubtful case, a radiologist with a 20-years job experience served as an independent and final arbiter. Clinically silent pulmonary radiological findings, specific for non-active tuberculosis, were recognized as heterogeneous, poorly defined fibro-nodular and/or fibrotic linear opacities and/or small dystrophic calcifications involving the apical and posterior segments of the upper lobes and the superior segments of the lower lobes. In any case of lung parenchymal lesions, once induced sputum was collected for *Mycobacterium bacillus* culture [1].

Venous blood for the quantiferon test (QFT) was collected from each subject before performing the tuberculin skin test (TST). The samples were collected by the trained personnel into two heparinised tubes from the kit: one tube with walls coated with ESAT-6, CFP-10, and TB7.7 antigens and the other tube containing pure physiological saline with phosphate buffer (negative control). The serum Quantiferon test (QFT) was performed according to the manufacturer’s instructions (Cellestis Ltd, Carnegie, Australia) as recommended by the manufacturer cut-off value for the positive result of interferon-γ concentration ≥0.35 IU/ml [8]. The tuberculin skin test was performed using the Mantoux method with 2 tuberculin units (0.1 ml) of a purified protein derivative RT23 (Statens Serum Institute, Copenhagen, Denmark). The TST reaction was scored as positive when the infiltration diameter was ≥10 mm. The TST was performed and read 72 h later by two very well-trained and well-experienced public health nurses. In the case of any inconsistency between the two nurses concerning the size of post-tuberculin infiltration, a well-experienced pulmonologist was asked for his/her opinion and the final result was agreed. Neither the laboratory worker performing the QFT nor the nurses conducting the TST knew results of the other test.

In the following five subgroups, sensitivity, specificity, accuracy, positive predictive value, negative predictive value, and positive and negative likelihood ratios for TST and QFT in the diagnosis of pulmonary radiological changes were assessed: TST ≥10 mm; TST≥15 mm; positive QFT; positive QFT and TST≥10 mm; positive QFT and TST≥15 mm.

The *T* test was used for the assessment of quantitative variables in two groups. The ANOVA analysis was used for the comparison of quantitative variables in more than 2 groups. In the case of qualitative variables, the Chi-squared and Fisher’s exact tests (for small numbers encountered) were used for the comparison of study subgroups. The multiple logistic regression model was applied for the assessment of influence of time from the date of TB contact, degrees of exposure, results of QFT, and accumulative results of QFT and TST on probability of the presence of latent post-tuberculosis radiological changes. The odds ratios were calculated for significant variables. The confidence interval (CI) was set at 95 % for significant differences.

## Results

A total of 96 healthcare workers of the Military Institute of Medicine declared a contact with tuberculosis in the past. Seven of them were excluded due to a history of active tuberculosis in the past; 1 was excluded due to recognized mycobacteriosis; further 5 individuals were not included as they did not meet the other inclusion criteria. Finally, 83 participants (64 females and 19 males), at a mean age of 43.5 (standard deviation - SD 10.9), were recruited to the study. Each participant was vaccinated against tuberculosis in the past as per the mandatory Polish vaccination schedule. Nobody had received anti-tuberculosis prophylaxis before including to the study. A mean period of employment in the healthcare facilities was 18.8 (SD 11.0) years. A mean time from the date of TB contact to the date of the present study inclusion was 13.3 (SD 9.4) years. Positive TST results were reported in 72 (86.8 %) participants while positive QFTs were observed in 27 (32.5 %) cases (Table 1). Screening chest scans (including 6 CT cases) revealed 23 (4 calcified nodules, 3 pleural thickenings, and 16 fibrous scarrings) radiological changes specific for non-active tuberculosis in 18 (21.7 %) participants: 15 (83.3 %) of them were TST positive (≥10 mm), and 13 (72.2 55.6 %) patients had positive QFT results. No pulmonary opacities were observed in 57 (79.2 %) out of 72 participants with the positive TST results (≥10 mm) and in 14 (51.9 %) out of 27 patients with the positive QFT results. Radiological opacities suggestive of latent tuberculosis were observed in 5 patients with negative QFT and in 3 cases with negative TST results (Table 1). A negative result of sputum culture was confirmed in each subject with radiological changes. Among the groups with positive QFTs and with or without radiological changes, no statistically significant differences between the interferon levels were found: 1.98 (CI: 0.5–4.3) vs. 2.0 (CI: 0.7–3.5) IU/ml; t test, *p* = 0.9. Time from TB contact in people with and without radiological opacities subtly diverged and amounted accordingly 17.3 (SD 11.0) and 12.2 (SD 8.7) years - the difference was not statistically significant (*p* = 0.052).

**Table 1:** Characteristics of the subgroups with and without silent chest X-ray findings

Characteristics *n* = 83 (64 females, 19 males)	Participants with pulmonary lesions *n* = 18 (15 females, 3 males)	Participants without pulmonary lesions *n* = 65 (49 females, 16 males)
Age mean (SD) yr	46.6 (11.3)	42.7 (10.8)*
Time from TB contact, mean (SD) yr	17.0 (10.8)	12.2 (8.7)**
Circle of contact, *n* (% of whole group)		
Inner	9 (10.8)	12 (14.5)
Middle	5 (6.0)	28 (33.7)
Outer	4 (4.8)	25 (30.1)
TST ≥10 mm, *n* (% of whole group)	15 (18.1)	57 (68.7)
TST <10 mm, *n* (% of whole group)	3 (3.6)	8 (9.6)
TST ≥15 mm, *n* (% of whole group)	11 (13.3)	36 (43.4)
TST <15 mm, *n* (% of whole group)	7 (8.4)	29 (34.9)
QFT positive, *n* (% of whole group)	13 (15.7)	14 (16.9)
QFT negative, *n* (% of whole group)	5 (6.0)	51 (61.4)
QFT positive and TST ≥10, *n* (% of whole group)	10 (12.1)	13 (15.7)
QFT negative and TST <10 mm, *n* (% of whole group)	8 (9.6)	52 (62.3)
QFT positive and TST ≥ 15 mm, * n* (% of whole group)	11 (13.3)	35 (42.2)
QFT negative and TST < 15 mm, *n* (% of whole group)	7 (8.4)	30 (36.1)

* p value = 0.2; ** p value = 0.052

It was observed that the positive results of QFT only and the simultaneously positive QFT and TST ≥10 mm were associated with the highest positive likelihood ratio and a positive predictive value of clinically silent chest X-ray findings suggestive of latent tuberculosis (Table 2).

**Table 2:** Usefulness of QFT and TST in prediction of clinically silent chest X-ray opacities characteristic for tuberculosis

	TST ≥ 10 mm	TST ≥15 mm	QFT positive	QFT positive and TST ≥10 mm	QFT positive and TST ≥15 mm
Sensitivity %	83.3 (60.8–94.2)	61.1 (38.6–79.7)	72.9 (49.1–87.5)	55.6 (33.7–75.4)	61.1 (38.6–79.7)
Specificity %	12.3 (6.4–22.5)	44.6 (33.2–56.7)	78.5 (67.0–86.7)	80.0 (68.7–87.9)	46.2 (34.6–58.2)
Accuracy %	28.9 (20.3–39.4)	48.2 (37.8–58.8)	77.1 (67.0–84.8)	74.7 (64.4–82.8)	49.4 (38.9–59.9)
Negative predictive value %	72.7 (43.4–90.3)	80.1 (65.0–90,3)	91.1 (80.7–96.1)	86.7 (75.8–93.1)	81.1 (65.8–90.5)
Positive predictive value %	20.8 (13.1–31.2)	23.4 (13.6–37.2)	48.2 (30.7–66.0)	43.5 (25.6–63.2)	23.9 (13.9–37.9)
Positive likelihood ratio LR+	1.0 (0.8–1.2)	1.1 (0.7–1.7)	3.4 (1.9–5.8)	2.8 (1.5–55.3)	1.1 (0.7–1.8)
Negative likelihood ratio LR-	1.4 (0.4–4.6)	0.9 (4.6–16.5)	0.3 (0.2–0.7)	0.6 (0.3–0.9)	0.8 (0.5–1.6)

According to the degrees of exposure defined by European consensus [1], among 83 participants, there were 21 (25.3 %) HCWs around the 1st (inner) circle of contacts, 33 (39.8 %) HCWs around the 2nd (middle) circle of contacts, and 29 (34.9 %) participants around the 3rd (outer) circle of contacts (Table 1). Positive QFT results were observed in 61.9, 24.2, and 20.7 % of the contact patients, respectively. Simultaneously, positive QFTs and TSTs ≥10 mm were reported in 57.1, 24.2, and 10.3 % of the participants in the inner, middle, and outer circles of contacts (Table 3). No statistically significant differences in the interferon level between the QFT positive participants in the 1st, 2nd, and 3rd circles were found: 1.8 (CI: 0.7–3.8) vs. 3.0 (CI: 0.8–4.9) IU/ml vs. 1.7 (CI: 1.0–3.1); ANOVA, p = 0.1.

**Table 3:** QFT, TST, and chest X-ray results in relation to the degree of exposure

	**Circle 1** *n* = 21	**Circle 2** *n* = 33	**Circle 3** *n* = 29	**Overall** *n* = 83	
**QFT positive**					
Yes	13 (61.9 %)	8 (24.2 %)	6 (20.7 %)	27 (32.5 %)	Circle 1 vs. 2; *p* = 0.006*
No	8 (38.1 %)	25 (75.8 %)	23 (79.3 %)	56 (67.5 %)	Circle 1 vs. 3; *p* = 0.003* Circle 2 vs. 3; *p* = 0.7
TST positive					
Yes	20 (95.2 %)	29 (87.9 %)	23 (79.3 %)	72 (86.7 %)	Circle 1 vs. 2; *p* = 0.4
No	1 (4.8 %)	4 (12.1 %)	6 (20.7 %)	11 (13.3 %)	Circle 1 vs. 3; *p* = 0.1 Circle 2 vs. 3; *p* = 0.4
QFT positive and TST ≥10 mm					
Yes	12 (57.1 %)	8 (24.2 %)	3 (10.3 %)	23 (27.7 %)	Circle 1 vs. 2; *p* = 0.02*
No	9 (42.9 %)	25 (75.8 %)	26 (89.7 %)	60 (72.3 %)	Circle 1 vs. 3; *p* = 0.0004* Circle 2 vs. 3; *p* = 0.2
Chest X-ray findings					
Present	9 (42.9 %)	5 (15.1 %)	4 (13.8 %)	18 (21.7 %)	Circle 1 vs. 2; *p* = 0.02*
Absent	12 (57.1 %)	28 (84.9 %)	25 (86.2 %)	65 (78.3 %)	Circle 1 vs. 3; *p* = 0.02* Circle 2 vs. 3; *p* = 0.9

* Statistically significant *p* value

In the contact circle 1 patients, significantly more frequent “clinically silent” pulmonary opacities in the radiographs (Table 3), positive QFTs and simultaneously positive QFTs and TSTs ≥10 mm were reported. There was no significant differences in percentage of patients with positive TST in relation to circle of TB contact (Table 3). In the log-linear analysis, the only statistically significant variable related to increased risk of late “clinically silent” radiological findings in the lung parenchyma specific for tuberculosis was the positive QFT result. Closeness and intensity of the contact as well as the simultaneous positive results of TST and QFT did not significantly increase the risk of pulmonary parenchymal opacities. Similarly, time from the date of TB contact had no influence on probability of presence of radiological opacities (Table 4).

**Table 4:** Results of the log-linear analysis for predictors of presence of late clinically silent pulmonary opacities (logistic regression model; *χ* ^2^ = 18.7, overall *p* = 0.0009)

Variable	Odds ratio	95 % CI	*p* value
Circle of contact			
1st vs. 2nd and 3rd	0.5	0.2–1.3	0.1
QFT positive			
Yes vs. no	8.3	2.5–27.8	0.005
QFT positive and TST ≥10 mm			
Yes vs. no	0.3	0.07–1.2	0.08
Time from TB contact	1.04	0.9–1.1	0.2

## Discussion

Latent tuberculosis infection is assumed to be a probable *M. tuberculosis complex* infection based on a positive TST and/or a positive QFT result with no clinical, bacteriological, or radiological symptoms of disease [1, 9]. However, it is believed that in patients with latent TB infection, “clinically silent” calcified, fibrotic, and fibro-nodular pulmonary lesions suggestive of previous pulmonary tuberculosis may be present [1]. In clinical practice, available tests that assess latent *M. tuberculosis* infection actually evaluate an adaptive immune response against mycobacterial antigens, and they are not able to distinguish between immunological memory and an ongoing effector T-cell response. Currently, there are no diagnostic tools that ensure direct infection identification, i.e., confirmation of TB bacteria presence in the body. The highest risk of tuberculosis occurs immediately after the contact with an infected person and it decreases gradually over 2 years. After this period, the risk maintains at a stable, low level; it is approximately one case per 1,000 person-years [10, 11]. According to the currently applicable strategy of management of individuals who have had a close contact with a TB patient, a TST and/or IGRA as well as a chest X-ray must be performed as soon as possible after the exposure and then after a window period, i.e., 8 weeks following the last contact with the index case during the period of infectiousness [1]. Nosocomial transmission of *M. tuberculosis* is well known, and the healthcare staff is considered a group at a higher risk of close contact with TB patients [3–7]. In our previous study, we found positive TSTs in 63.6 % and positive QFTs in 20.5 % of the health care professionals who declared any (even accidental) contact with tuberculosis in the past [7]. We also revealed that in the Polish society, a high probability of false-positive TSTs due to post-vaccination reactions made the tuberculin skin test a less useful diagnostic method than IGRAs for the evaluation of LTBI [7]. As opposed to the TST, positive QFT results were significantly related to some independent occupational and non-occupational risk factors, such as an older age, a lower education level, or a period of employment in the health care field of Polish HCWs [7]. In the present study, due to a restrictive classification of contacts around the index case according to the degree of exposure consistent with the European recommendations and inclusion of participants subjected to screening tests after a TB contact in the past into the analysis, proportions of positive TST and QFT results in the study group were higher: 86.8 and 32.5 %, respectively. In our study, late radiological pulmonary findings suggestive of previous tuberculosis were reported in 18 (21.7 %) out of 83 HCWs who declared a contact with tuberculosis in the past. The changes were not found during radiological examinations performed within 2 months to 2 years following the contact. In the literature, a poor usefulness of screening chest radiological examinations for tuberculosis detection in countries with a low TB prevalence among asymptomatic individuals is emphasized [12, 13]. Eisenberg [12], while studying asymptomatic HCWs with positive TSTs in the pre-employment setting, found radiological changes suggestive of previous tuberculosis in only 6.1 % of the subjects. In countries with a high TB prevalence, radiological findings suggestive of previous, non-active tuberculosis are reported in more than 60 % of HCWs with a positive TST or QFT result [14]. Moreover, in some studies conducted in high TB incidence settings, no difference in positive rates of TST as well as QFT was revealed between people with and without X-ray chest lesions suggestive of old-healed TB [14]. On the other hand, the results of another study denote higher positive rates of TST and QFT among participants with silent post-tuberculosis pulmonary lesions than among people without old-healed opacities [15]. In our study, we found lesions specific for previous tuberculosis in 21.7 % of clinically asymptomatic HCWs who had been exposed to TB more than 10 years before. All participants with radiological changes had standard screening tuberculosis tests in the past soon after a contact with the disease, and none of them suffered from TB from the contact date until inclusion into the study. As in the case of positive QFT results, radiological changes were significantly more frequent in the participants who declared close and intense contact with tuberculosis patients (circle 1) compared to the participants in the middle and outer circles (Table 3). The degree of intensity of contact with the index case did not significantly affect proportions of positive TST results in the inner, middle, and outer circles: 95.2, 87.9, and 79.3 %, respectively. The results of the TSTs showed a markedly lower positive predictive value and positive likelihood ratio compared to the positive results of the QFTs with respect to a prognosis of post-tuberculosis lesions in the asymptomatic patients (Table 2). A lower diagnostic value of the TST clearly results from a great number of false-positive post-vaccination tuberculin test as in Poland, for many years until 2006, an obligatory schedule of vaccination against tuberculosis existed, involving even five BCG injections in the period from the birth to 18 years of age depending on the size of post-vaccination scar after the 1st year of life and diameters of post-tuberculin infiltrations at the age of 7, 12, and 18.

The positive QFT result in the log-linear analysis was the only relevant predictive factor of the risk of radiological findings specific for previous tuberculosis many years after the exposure (OR = 8.3; *p* = 0.005). In the log-linear analysis, closeness and intensity of the contact with a tuberculosis patient did not increase the risk of presence of lesions suggestive of previous tuberculosis following the period of standard screening tests related to TB exposure. In view of no effects of TB exposure intensity on the presence of radiological changes, a prognostic value of the positive QFTs can be a manifestation of phenotypic predisposition of certain individuals to morphological and immunological responses that lead to the initial immune priming and development of clinically silent radiological findings suggestive of previous tuberculosis many years following the exposure and normal screening results. A certain disadvantage of the study is the fact that we cannot definitely exclude the possibility that the participants had more unaware and not reported contacts with tuberculosis following the first exposure and that they had self-healed TB. However, none of the participants reported any typical TB symptoms to occur since the date of first contact, such as chronic cough, long-term subfebrile temperature, weight loss, or haemoptysis. Other disadvantages of the study are exclusion of people with immunosuppressive diseases and indeterminate result of QFT. Summing up, a positive QFT result is associated with an 8.3 odds ratio of clinically silent lesions in the lung parenchyma suggestive of previous pulmonary tuberculosis in asymptomatic individuals who have never had active TB, and the results of their radiographic tests performed within the recommended post-exposure period have been normal. Our findings suggest that in some people, there could exist a phenotypic immune predisposition for development of radiological opacities after exposure to tuberculosis, and about one-half of the patients with a positive result on their QFT, who have the correct result from the chest X-ray exam during the primary period after contact with tuberculosis could develop silent radiological symptoms of tuberculosis many years after the date of exposure (positive predictive value–48.2 %). Moreover, negative results from the QFT performed many years after contact with tuberculosis seem to be strong evidence that there are no old-healed post-tuberculosis lesions in the lung parenchyma (negative predictive value–91.1 %). These results compel to afterthought whether people with positive QFT, particularly if they had close contact with tuberculosis, should cover more careful and extended medical care after contact with tuberculosis, and whether such patients could have X-ray follow-up exams done many years after contact with tuberculosis. However, further studies are needed for confirmation of these guesses.
